# Good educators and orphans: the case of direct observation and feedback

**DOI:** 10.1111/medu.13835

**Published:** 2019-03-15

**Authors:** Chris BT Rietmeijer, Pim W Teunissen

**Affiliations:** ^1^ Amsterdam University medical centers location VUmc Department of General practice and elderly care medicine Amsterdam the Netherlands; ^2^ School of Health Professions Education (SHE) Faculty of Health, Medicine and Life Sciences (FHML) Maastricht University Maastricht the Netherlands

## Abstract

The authors propose the concept of orphaned competencies, those that are good for clinical education, but often are not taken up by good clinical educators. Prolonged direct observation is promoted as a way forward.

What do we expect from a good clinical educator? For anyone who thinks back to an inspiring (clinical) teacher, the answer to this question is likely to involve gentleness, skill, interest in the learner and, importantly, the habit of investing time and effort in teaching. This is in line with what Gibson and colleagues found in their review of research on clinical educators’ skills and qualities in allied health, published in this issue.^1^ The authors identify seven themes in educators’ skills and qualities and, importantly, highlight the overarching concept of taking time to perform the clinical educator role. These results resonate with comparable reviews in other domains, such as postgraduate medical education (PGME).^2^, ^3^, ^4^ Gibson and colleagues state that we have now reached a point at which we are well informed about learners’ and clinical educators’ perceptions of what good clinical education entails.^1^ Future research, in their opinion, should take new perspectives, such as by focusing on the effects of clinical educators’ efforts on learners’ progress or on patient care. We agree and would add an additional topic that requires attention: the varying realisation of what we know about being a good clinical educator, or supervisor, in day‐to‐day clinical education practice.

The authors highlight the overarching concept of taking time to perform the clinical educator role

We elaborate on this by zooming in on direct observation and feedback as central constituents of supervision. Direct observation is crucial for the purposes of feedback and assessment, as Kogan and colleagues describe in their guidelines on direct observation.^5^ Yet, as Kogan et al. also state, direct observation in medical education is often infrequent and of poor quality.^5^ We first discuss how recent research on optimising direct observation and feedback aligns with what we know about being a good clinical educator. Subsequently, we focus on when these standards for constructive direct observation and feedback are met in the workplace, and when they are not.

Engaging in direct observation and feedback, according to a growing body of research, is challenging for residents and supervisors. Residents struggle with unclear stakes, fears of assessment, credibility judgements regarding the observer, and issues around authenticity, autonomy and efficiency.^6^, ^7^, ^8^, ^9^, ^10^ In this literature, longitudinal training relationships are mentioned as providing a way forward.^5^, ^6^ Moreover, new perspectives on feedback suggest the importance of a longitudinal educational alliance between the resident and supervisor in which the resident likes and values the supervisor, and both agree on learning goals and the ways these can be achieved.^11^, ^12^ In this, feedback is part of an ongoing dialogue. Other research adds the power of the bi‐directionality of direct observation, meaning that the resident alternately observes and is observed by the supervisor;^13^ the resident and supervisor give one another feedback and both learn while working together.^14^ In essence, this literature on direct observation and feedback points to conditions for constructive educational situations that align with what we know about being a good clinical educator.^1^, ^3^ In this, the investing of time and effort is clearly a basic prerequisite.

Now that we have identified these conditions for constructive direct observation and feedback, the next question concerns when they are met in the workplace. The literature provides us with a clue. Watling and colleagues, in an interview study amongst residents from a number of medical specialties, found that ‘cultures observed what they valued’.^9^ Surgeons observed their residents at the operating table; psychiatrists observed residents engaged in history taking.^9^ With respect to these valued competencies, at least some of the basic conditions for constructive direct observation and feedback, like the investment of time and effort, seemed to be in place.^9^

If cultures observe what they value, they will, in consequence, pay less attention to what they value less.^9^, ^14^ This means that some competencies are less observed and receive less attention; we suggest calling these ‘orphaned competencies’. These may be competencies in which educators themselves have never been formally trained, such as teamwork, or new competencies relating to new developments, such as e‐health, in which seasoned clinical educators may not feel they are expert. Individual supervisors may feel that some competencies are less crucial or less indisputable, such as communication skills. Moreover, competencies such as physical examination may be orphaned because supervisors think that residents should have mastered them in medical school.^9^, ^14^

Some competencies are less observed and receive less attention; we suggest calling these ‘orphaned competencies’

One might argue that, in competency‐based medical education, attention to all competencies is ensured.^15^ In PGME settings many competencies are observed in different ways and with different tools, such as the mini‐clinical examination. However, currently, these observations are typically shaped as uni‐directional, brief assessments of learning, often performed by different observers.^16^, ^17^ This practice conflicts with the aforementioned conditions for constructive direct observation and feedback, which makes its value for learning questionable. This brings us to the conclusion that, if we want to be good clinical educators with regard to all competencies, including the orphaned ones, we have to find new ways to ensure constructive direct observation and feedback for all competencies.

If we want to be good clinical educators in all competencies, including the orphaned ones, we have to find new ways to ensure constructive direct observation and feedback

Recent research provides us with examples of such new ways. Voyer and colleagues^18^ investigated the delivery of feedback through extensive direct observation sessions of a few hours every 2 or 3 months, within a prolonged training relationship and devoid of summative assessment. First‐year internal residents were observed while performing usual clinical duties on typical internal medicine inpatient rotations. Residents valued the direct observation of their day‐to‐day work, which comprised many different activities that normally were not supervised.^18^ In general practice training, supervisors were found to initiate weekly bi‐directional direct observation sessions of 1–2 hours, in which they observed and demonstrated a broad spectrum of activities and competencies.^14^ Importantly, in both examples, the educational effort was not focused on any particular activity or competency but on the whole performance as a doctor. In this, many competencies became visible.^14^, ^18^

The educational effort was not focused on any particular activity or competency but on the whole performance as a doctor

Extensive direct observation sessions thus provide occasions for clinical education in all competencies. However, clinical educators’ awareness and recognition of orphaned competencies that present themselves in these patient care situations, and willingness to invest in them, are equally important. In this, supervisors will often be learners themselves and hence supervisors and residents work and learn together. As we know from Gibson et al.'s review,^1^ learners in allied health value collaborative learning with educators who are on a learning continuum themselves.

Extensive direct observation sessions thus provide occasions for clinical education in all competencies

To conclude, we propose the concept of orphaned competencies, for which potentially good clinical educators may fail to engage in good clinical education. The focus on situations in which clinical educators succeed in teaching all competencies and the situations in which some competencies are orphaned offers a direction for future research.
